# Dihydro­nium hexa­kis[bromido/chlorido(0.75/0.25)]dicadmate(II)–triphenyl­phosphine oxide (1/6)

**DOI:** 10.1107/S1600536809020388

**Published:** 2009-06-06

**Authors:** Kong Mun Lo, Seik Weng Ng

**Affiliations:** a Department of Chemistry, University of Malaya, 50603 Kuala Lumpur, Malaysia

## Abstract

In the salt, (H_3_O)_2_[Cd_2_Br_4.5_Cl_1.5_]·6C_18_H_15_OP, the hydro­nium cation forms short O—H⋯O hydrogen bonds to the O atoms of the triphenyl­phosphine oxide units. The centrosymmetric dinuclear anion has two halide atoms functioning in a bridging mode, which confers tetra­hedral coordination to the Cd atom. The three independent halide atoms are each a mixture of bromide and chloride; the occupancies of the Br atoms are 0.6434 (11), 0.7468 (11) and 0.8598 (11).

## Related literature

There is only one example of a [(Ph_3_PO)_3_·H_3_O]^+^ system: for the [Mo_6_Cl_14_]^2−^ salt, see: Kozhomuratova *et al.* (2007 ▶).
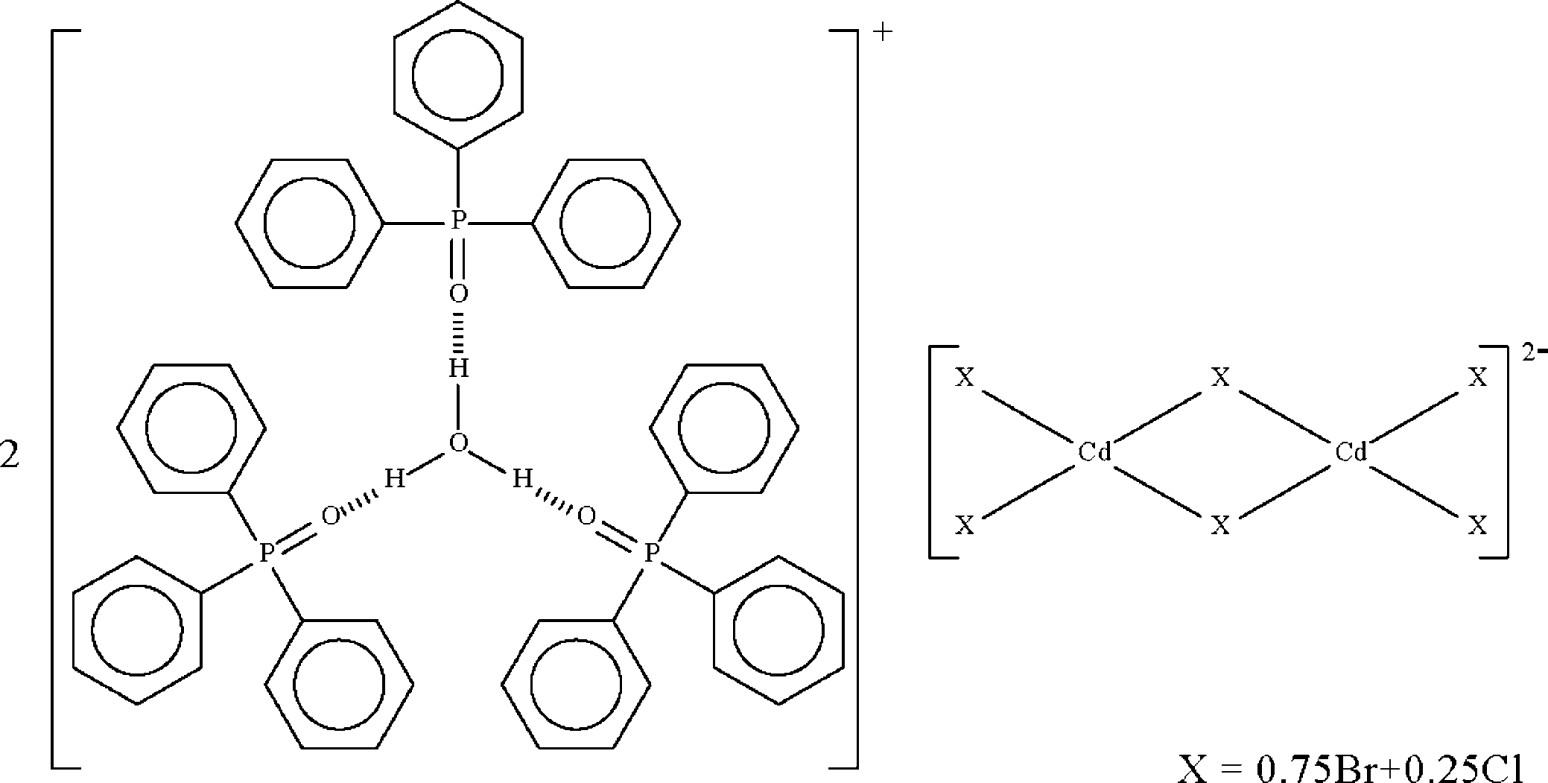


## Experimental

### 

#### Crystal data

(H_3_O)_2_[Cd_2_Br_4.5_Cl_1.5_]·6C_18_H_15_OP
*M* *_r_* = 2345.24
Triclinic, 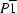

*a* = 11.1623 (2) Å
*b* = 15.0677 (3) Å
*c* = 16.0911 (3) Å
α = 75.499 (1)°
β = 81.013 (1)°
γ = 79.605 (1)°
*V* = 2559.57 (8) Å^3^
*Z* = 1
Mo *K*α radiation
μ = 2.36 mm^−1^
*T* = 133 K
0.20 × 0.10 × 0.05 mm

#### Data collection

Bruker SMART APEX diffractometer
Absorption correction: multi-scan (*SADABS*; Sheldrick, 1996 ▶) *T*_min_ = 0.650, *T*_max_ = 0.891
21253 measured reflections
11527 independent reflections
8297 reflections with *I* > 2σ(*I*)
*R*_int_ = 0.028

#### Refinement

*R*[*F* ^2^ > 2σ(*F* ^2^)] = 0.036
*wR*(*F* ^2^) = 0.096
*S* = 0.98
11527 reflections
601 parameters
7 restraints
H atoms treated by a mixture of independent and constrained refinement
Δρ_max_ = 0.93 e Å^−3^
Δρ_min_ = −0.56 e Å^−3^

### 

Data collection: *APEX2* (Bruker, 2007 ▶); cell refinement: *SAINT* (Bruker, 2007 ▶); data reduction: *SAINT*; program(s) used to solve structure: *SHELXS97* (Sheldrick, 2008 ▶); program(s) used to refine structure: *SHELXL97* (Sheldrick, 2008 ▶); molecular graphics: *X-SEED* (Barbour, 2001 ▶); software used to prepare material for publication: *publCIF* (Westrip, 2009 ▶).

## Supplementary Material

Crystal structure: contains datablocks global, I. DOI: 10.1107/S1600536809020388/tk2468sup1.cif

Structure factors: contains datablocks I. DOI: 10.1107/S1600536809020388/tk2468Isup2.hkl

Additional supplementary materials:  crystallographic information; 3D view; checkCIF report

## Figures and Tables

**Table 1:** Hydrogen-bond geometry (Å, °)

*D*—H⋯*A*	*D*—H	H⋯*A*	*D*⋯*A*	*D*—H⋯*A*
O4—H41⋯O1	0.85 (1)	1.62 (1)	2.472 (3)	177 (4)
O4—H42⋯O2	0.85 (1)	1.63 (1)	2.471 (3)	176 (3)
O4—H43⋯O3	0.85 (1)	1.63 (1)	2.481 (3)	177 (3)

**Fig. 1.**
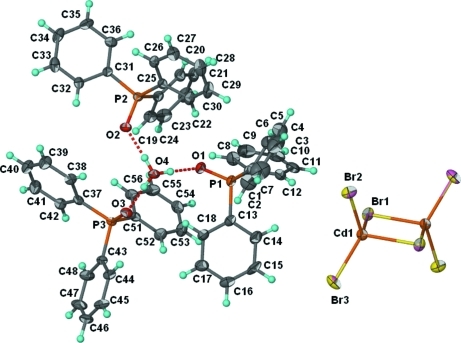
Thermal ellipsoid plot (Barbour, 2001) of 2[Ph3PO.(OH3)] [Cd2Br4.5Cl1.5] at the 70% probability level. Hydrogen atoms are drawn as spheres of arbitrary radius. Hydrogen bonds are drawn as dashed lines. The Br/Cl disorder is not shown.

## supplementary crystallographic information

### Experimental

Cadmium chloride dihydrate (0.22 g, 1 mmol) was dissolved in water (10 ml); triphenylphosphine dibromide (0.8 g, 2 mmol) was dissolved in ethanol (90 ml). The two solutions were mixed and then heated for 1 h. Slow evaporation of the filtrate gave colorless, irregularly-shaped crystals.

### Refinement

Hydrogen atoms were placed at calculated positions (C–H 0.95 Å) and were treated as riding on their parent atoms, with *U*(H) set to 1.2*U*_eq_(C). The acid H-atoms were located in a difference Fourier map, and were refined with a distance restraint of O–H 0.84±0.01 Å; their isotropic temperature factors were refined.

The three independent halogen atoms are each a mixture of bromide and chloride atoms. The total bromide occupancy refined to nearly 2.25; this was then set as exactly 2.25. The occupancies of the Br1, Br2 and Br3 atoms are 0.6434 (11), 0.7468 (11) and 0.8598 (11).

### Crystal data

**Table:**

(H_3_O)_2_[Cd_2_Br_4.5_Cl_1.5_]·6C_18_H_15_OP	*Z* = 1
*M**_r_* = 2345.24	*F*(000) = 1177
Triclinic, *P*1	*D*_x_ = 1.521 Mg m^−^^3^
Hall symbol: -P 1	Mo *K*α radiation, λ = 0.71073 Å
*a* = 11.1623 (2) Å	Cell parameters from 5205 reflections
*b* = 15.0677 (3) Å	θ = 2.2–27.5°
*c* = 16.0911 (3) Å	µ = 2.36 mm^−^^1^
α = 75.499 (1)°	*T* = 133 K
β = 81.013 (1)°	Irregular block, colorless
γ = 79.605 (1)°	0.20 × 0.10 × 0.05 mm
*V* = 2559.57 (8) Å^3^	

### Data collection

**Table:**

Bruker SMART APEX diffractometer	11527 independent reflections
Radiation source: fine-focus sealed tube	8297 reflections with *I* > 2σ(*I*)
graphite	*R*_int_ = 0.028
ω scans	θ_max_ = 27.5°, θ_min_ = 1.4°
Absorption correction: multi-scan (*SADABS*; Sheldrick, 1996)	*h* = −13→14
*T*_min_ = 0.650, *T*_max_ = 0.891	*k* = −19→19
21253 measured reflections	*l* = −20→20

### Refinement

**Table:**

Refinement on *F*^2^	Primary atom site location: structure-invariant direct methods
Least-squares matrix: full	Secondary atom site location: difference Fourier map
*R*[*F*^2^ > 2σ(*F*^2^)] = 0.036	Hydrogen site location: inferred from neighbouring sites
*wR*(*F*^2^) = 0.096	H atoms treated by a mixture of independent and constrained refinement
*S* = 0.98	*w* = 1/[σ^2^(*F*_o_^2^) + (0.0497*P*)^2^] where *P* = (*F*_o_^2^ + 2*F*_c_^2^)/3
11527 reflections	(Δ/σ)_max_ = 0.001
601 parameters	Δρ_max_ = 0.93 e Å^−^^3^
7 restraints	Δρ_min_ = −0.56 e Å^−^^3^

### Special details

**Table:**

Geometry. All esds (except the esd in the dihedral angle between two l.s. planes) are estimated using the full covariance matrix. The cell esds are taken into account individually in the estimation of esds in distances, angles and torsion angles; correlations between esds in cell parameters are only used when they are defined by crystal symmetry. An approximate (isotropic) treatment of cell esds is used for estimating esds involving l.s. planes.

### Fractional atomic coordinates and isotropic or equivalent isotropic displacement parameters (Å^2^)

**Table:**

	*x*	*y*	*z*	*U*_iso_*/*U*_eq_	Occ. (<1)
Cd1	0.49843 (2)	0.596709 (15)	0.544155 (16)	0.02993 (7)	
Br1	0.31897 (4)	0.52389 (3)	0.50664 (3)	0.03634 (15)	0.6434 (11)
Br2	0.47394 (4)	0.56628 (3)	0.70646 (3)	0.04195 (15)	0.7468 (11)
Br3	0.51554 (4)	0.75339 (3)	0.44674 (3)	0.04616 (15)	0.8598 (11)
Cl1	0.31897 (4)	0.52389 (3)	0.50664 (3)	0.03634 (15)	0.36
Cl2	0.47394 (4)	0.56628 (3)	0.70646 (3)	0.04195 (15)	0.25
Cl3	0.51554 (4)	0.75339 (3)	0.44674 (3)	0.04616 (15)	0.14
P1	0.77902 (8)	0.75651 (6)	0.79120 (6)	0.03052 (19)	
P2	1.04703 (7)	0.76284 (5)	1.06470 (5)	0.02452 (17)	
P3	1.10446 (7)	1.03723 (5)	0.71506 (5)	0.02213 (17)	
O1	0.8679 (2)	0.78431 (15)	0.83765 (15)	0.0373 (6)	
O2	1.0201 (2)	0.86060 (14)	1.01185 (13)	0.0307 (5)	
O3	0.98970 (18)	1.02236 (14)	0.77588 (13)	0.0285 (5)	
O4	0.8825 (2)	0.91749 (16)	0.89733 (14)	0.0289 (5)	
H41	0.876 (4)	0.8710 (17)	0.878 (2)	0.16 (3)*	
H42	0.927 (2)	0.898 (2)	0.9383 (13)	0.038 (11)*	
H43	0.920 (3)	0.9536 (19)	0.8569 (14)	0.052 (12)*	
C1	0.6446 (3)	0.7263 (2)	0.8628 (2)	0.0353 (8)	
C2	0.5511 (4)	0.7959 (3)	0.8795 (3)	0.0566 (11)	
H2	0.5522	0.8574	0.8458	0.068*	
C3	0.4570 (4)	0.7774 (4)	0.9440 (3)	0.0773 (15)	
H3	0.3938	0.8260	0.9548	0.093*	
C4	0.4536 (4)	0.6895 (4)	0.9928 (3)	0.0697 (14)	
H4	0.3879	0.6767	1.0372	0.084*	
C5	0.5457 (5)	0.6194 (4)	0.9774 (3)	0.0672 (13)	
H5	0.5444	0.5583	1.0121	0.081*	
C6	0.6409 (4)	0.6371 (3)	0.9114 (2)	0.0511 (10)	
H6	0.7029	0.5882	0.8999	0.061*	
C7	0.8528 (3)	0.6591 (2)	0.7477 (2)	0.0332 (8)	
C8	0.9792 (3)	0.6371 (3)	0.7442 (3)	0.0462 (9)	
H8	1.0241	0.6719	0.7666	0.055*	
C9	1.0404 (4)	0.5647 (3)	0.7082 (3)	0.0560 (11)	
H9	1.1270	0.5493	0.7069	0.067*	
C10	0.9760 (5)	0.5152 (3)	0.6743 (2)	0.0549 (11)	
H10	1.0183	0.4662	0.6487	0.066*	
C11	0.8506 (4)	0.5363 (2)	0.6773 (2)	0.0502 (11)	
H11	0.8068	0.5017	0.6537	0.060*	
C12	0.7870 (4)	0.6077 (2)	0.7145 (2)	0.0389 (8)	
H12	0.7002	0.6214	0.7173	0.047*	
C13	0.7263 (3)	0.8497 (2)	0.7052 (2)	0.0313 (7)	
C14	0.6436 (3)	0.8396 (3)	0.6530 (3)	0.0484 (10)	
H14	0.6134	0.7824	0.6623	0.058*	
C15	0.6057 (4)	0.9135 (3)	0.5873 (3)	0.0512 (11)	
H15	0.5499	0.9062	0.5515	0.061*	
C16	0.6466 (3)	0.9969 (3)	0.5729 (2)	0.0412 (9)	
H16	0.6193	1.0470	0.5277	0.049*	
C17	0.7274 (3)	1.0074 (3)	0.6243 (2)	0.0449 (9)	
H17	0.7564	1.0650	0.6149	0.054*	
C18	0.7670 (3)	0.9338 (2)	0.6903 (2)	0.0375 (8)	
H18	0.8230	0.9417	0.7256	0.045*	
C19	1.1078 (3)	0.6836 (2)	0.9960 (2)	0.0301 (7)	
C20	1.0993 (3)	0.5894 (2)	1.0225 (2)	0.0347 (8)	
H20	1.0559	0.5655	1.0768	0.042*	
C21	1.1544 (3)	0.5306 (2)	0.9692 (3)	0.0419 (9)	
H21	1.1482	0.4664	0.9868	0.050*	
C22	1.2189 (4)	0.5656 (3)	0.8899 (3)	0.0481 (10)	
H22	1.2572	0.5249	0.8538	0.058*	
C23	1.2275 (4)	0.6582 (3)	0.8635 (2)	0.0480 (10)	
H23	1.2718	0.6815	0.8093	0.058*	
C24	1.1713 (3)	0.7180 (2)	0.9162 (2)	0.0377 (8)	
H24	1.1763	0.7824	0.8977	0.045*	
C25	0.9143 (3)	0.72130 (19)	1.1300 (2)	0.0255 (7)	
C26	0.8974 (3)	0.7105 (2)	1.2190 (2)	0.0304 (7)	
H26	0.9599	0.7209	1.2477	0.036*	
C27	0.7889 (3)	0.6842 (2)	1.2662 (2)	0.0397 (8)	
H27	0.7774	0.6768	1.3272	0.048*	
C28	0.6984 (3)	0.6691 (2)	1.2248 (3)	0.0460 (10)	
H28	0.6247	0.6505	1.2572	0.055*	
C29	0.7147 (3)	0.6810 (3)	1.1358 (3)	0.0473 (10)	
H29	0.6515	0.6709	1.1074	0.057*	
C30	0.8210 (3)	0.7070 (2)	1.0880 (2)	0.0371 (8)	
H30	0.8312	0.7153	1.0269	0.045*	
C31	1.1628 (3)	0.7585 (2)	1.13150 (19)	0.0246 (6)	
C32	1.2048 (3)	0.8411 (2)	1.1285 (2)	0.0276 (7)	
H32	1.1702	0.8974	1.0931	0.033*	
C33	1.2963 (3)	0.8413 (2)	1.1767 (2)	0.0390 (9)	
H33	1.3242	0.8979	1.1745	0.047*	
C34	1.3477 (3)	0.7599 (3)	1.2282 (2)	0.0419 (9)	
H34	1.4105	0.7607	1.2614	0.050*	
C35	1.3079 (3)	0.6769 (3)	1.2315 (2)	0.0379 (8)	
H35	1.3437	0.6209	1.2666	0.046*	
C36	1.2160 (3)	0.6763 (2)	1.1834 (2)	0.0330 (8)	
H36	1.1887	0.6194	1.1856	0.040*	
C37	1.2285 (3)	1.04350 (19)	0.7715 (2)	0.0245 (7)	
C38	1.2051 (3)	1.0362 (2)	0.8601 (2)	0.0302 (7)	
H38	1.1259	1.0267	0.8892	0.036*	
C39	1.2966 (3)	1.0426 (2)	0.9059 (2)	0.0397 (8)	
H39	1.2801	1.0374	0.9665	0.048*	
C40	1.4112 (3)	1.0564 (3)	0.8646 (2)	0.0420 (9)	
H40	1.4735	1.0615	0.8964	0.050*	
C41	1.4359 (3)	1.0629 (3)	0.7761 (2)	0.0412 (9)	
H41A	1.5156	1.0714	0.7475	0.049*	
C42	1.3450 (3)	1.0571 (2)	0.7303 (2)	0.0340 (8)	
H42A	1.3618	1.0624	0.6697	0.041*	
C43	1.0715 (3)	1.14445 (19)	0.63665 (19)	0.0255 (7)	
C44	0.9536 (3)	1.1932 (2)	0.6446 (2)	0.0297 (7)	
H44	0.8934	1.1683	0.6886	0.036*	
C45	0.9236 (4)	1.2784 (2)	0.5885 (2)	0.0397 (9)	
H45	0.8427	1.3112	0.5936	0.048*	
C46	1.0109 (4)	1.3149 (2)	0.5258 (2)	0.0466 (10)	
H46	0.9911	1.3740	0.4886	0.056*	
C47	1.1273 (4)	1.2663 (3)	0.5167 (3)	0.0542 (11)	
H47	1.1868	1.2915	0.4723	0.065*	
C48	1.1586 (3)	1.1809 (2)	0.5717 (2)	0.0425 (9)	
H48	1.2390	1.1477	0.5649	0.051*	
C51	1.1527 (3)	0.9460 (2)	0.65886 (19)	0.0248 (7)	
C52	1.0762 (3)	0.9363 (2)	0.6022 (2)	0.0364 (8)	
H52	1.0071	0.9821	0.5891	0.044*	
C53	1.0996 (3)	0.8607 (2)	0.5647 (2)	0.0417 (9)	
H53	1.0463	0.8541	0.5268	0.050*	
C54	1.2010 (3)	0.7948 (2)	0.5830 (2)	0.0383 (8)	
H54	1.2172	0.7425	0.5579	0.046*	
C55	1.2784 (3)	0.8046 (2)	0.6371 (2)	0.0417 (9)	
H55	1.3492	0.7599	0.6481	0.050*	
C56	1.2547 (3)	0.8790 (2)	0.6762 (2)	0.0336 (8)	
H56	1.3079	0.8842	0.7148	0.040*	

### Atomic displacement parameters (Å^2^)

**Table:**

	*U*^11^	*U*^22^	*U*^33^	*U*^12^	*U*^13^	*U*^23^
Cd1	0.02838 (13)	0.02682 (13)	0.03454 (15)	−0.00040 (9)	−0.00221 (10)	−0.01039 (10)
Br1	0.0245 (2)	0.0356 (2)	0.0516 (3)	0.00557 (16)	−0.00932 (19)	−0.0189 (2)
Br2	0.0390 (3)	0.0480 (3)	0.0349 (3)	0.00852 (18)	−0.00424 (18)	−0.0130 (2)
Br3	0.0531 (3)	0.0293 (2)	0.0501 (3)	−0.01052 (17)	0.0123 (2)	−0.00627 (18)
Cl1	0.0245 (2)	0.0356 (2)	0.0516 (3)	0.00557 (16)	−0.00932 (19)	−0.0189 (2)
Cl2	0.0390 (3)	0.0480 (3)	0.0349 (3)	0.00852 (18)	−0.00424 (18)	−0.0130 (2)
Cl3	0.0531 (3)	0.0293 (2)	0.0501 (3)	−0.01052 (17)	0.0123 (2)	−0.00627 (18)
P1	0.0294 (5)	0.0313 (4)	0.0342 (5)	−0.0064 (3)	−0.0102 (4)	−0.0082 (4)
P2	0.0319 (4)	0.0191 (4)	0.0227 (4)	−0.0026 (3)	−0.0059 (3)	−0.0042 (3)
P3	0.0224 (4)	0.0204 (4)	0.0212 (4)	−0.0015 (3)	−0.0004 (3)	−0.0027 (3)
O1	0.0388 (14)	0.0324 (12)	0.0453 (15)	−0.0043 (10)	−0.0203 (11)	−0.0088 (11)
O2	0.0424 (14)	0.0239 (11)	0.0248 (12)	−0.0014 (9)	−0.0087 (10)	−0.0032 (9)
O3	0.0240 (11)	0.0293 (11)	0.0265 (12)	−0.0031 (9)	0.0027 (9)	−0.0002 (9)
O4	0.0318 (13)	0.0282 (12)	0.0232 (12)	−0.0044 (10)	−0.0039 (10)	0.0009 (10)
C1	0.037 (2)	0.048 (2)	0.0265 (18)	−0.0140 (16)	−0.0096 (15)	−0.0085 (16)
C2	0.045 (2)	0.058 (3)	0.062 (3)	−0.0005 (19)	0.004 (2)	−0.016 (2)
C3	0.054 (3)	0.105 (4)	0.065 (3)	−0.003 (3)	0.011 (2)	−0.024 (3)
C4	0.046 (3)	0.119 (5)	0.044 (3)	−0.022 (3)	−0.001 (2)	−0.015 (3)
C5	0.077 (3)	0.082 (3)	0.043 (3)	−0.040 (3)	−0.003 (2)	0.002 (2)
C6	0.060 (3)	0.057 (3)	0.036 (2)	−0.016 (2)	−0.0075 (19)	−0.0042 (19)
C7	0.043 (2)	0.0297 (17)	0.0268 (18)	−0.0061 (14)	−0.0080 (15)	−0.0034 (14)
C8	0.041 (2)	0.043 (2)	0.055 (3)	−0.0032 (16)	−0.0058 (18)	−0.0131 (19)
C9	0.054 (3)	0.050 (2)	0.053 (3)	0.011 (2)	−0.002 (2)	−0.007 (2)
C10	0.090 (4)	0.035 (2)	0.032 (2)	0.002 (2)	−0.002 (2)	−0.0052 (17)
C11	0.094 (4)	0.0259 (19)	0.033 (2)	−0.015 (2)	−0.015 (2)	−0.0005 (16)
C12	0.053 (2)	0.0317 (18)	0.033 (2)	−0.0150 (16)	−0.0104 (17)	−0.0006 (15)
C13	0.0230 (17)	0.0373 (18)	0.0339 (19)	0.0008 (13)	−0.0084 (14)	−0.0096 (15)
C14	0.045 (2)	0.040 (2)	0.065 (3)	0.0020 (17)	−0.030 (2)	−0.013 (2)
C15	0.045 (2)	0.062 (3)	0.053 (3)	0.0124 (19)	−0.0332 (19)	−0.022 (2)
C16	0.036 (2)	0.047 (2)	0.035 (2)	0.0091 (16)	−0.0091 (16)	−0.0056 (17)
C17	0.041 (2)	0.049 (2)	0.039 (2)	−0.0114 (17)	−0.0064 (17)	0.0052 (18)
C18	0.037 (2)	0.045 (2)	0.0312 (19)	−0.0132 (16)	−0.0108 (15)	−0.0003 (16)
C19	0.0349 (18)	0.0274 (16)	0.0304 (18)	−0.0001 (13)	−0.0098 (14)	−0.0103 (14)
C20	0.039 (2)	0.0289 (17)	0.040 (2)	−0.0037 (14)	−0.0050 (16)	−0.0141 (15)
C21	0.040 (2)	0.0305 (18)	0.063 (3)	0.0009 (15)	−0.0147 (19)	−0.0238 (18)
C22	0.047 (2)	0.054 (2)	0.049 (2)	0.0075 (18)	−0.0099 (19)	−0.030 (2)
C23	0.056 (3)	0.053 (2)	0.033 (2)	0.0010 (19)	−0.0001 (18)	−0.0168 (18)
C24	0.045 (2)	0.0380 (19)	0.0288 (19)	−0.0012 (15)	−0.0023 (16)	−0.0105 (16)
C25	0.0270 (17)	0.0191 (14)	0.0319 (18)	0.0009 (12)	−0.0064 (13)	−0.0099 (13)
C26	0.0318 (18)	0.0304 (17)	0.0327 (19)	−0.0055 (13)	−0.0053 (14)	−0.0127 (14)
C27	0.037 (2)	0.043 (2)	0.039 (2)	−0.0089 (16)	0.0055 (16)	−0.0129 (17)
C28	0.0265 (19)	0.044 (2)	0.072 (3)	−0.0051 (15)	0.0046 (18)	−0.027 (2)
C29	0.031 (2)	0.050 (2)	0.073 (3)	−0.0054 (16)	−0.0127 (19)	−0.031 (2)
C30	0.033 (2)	0.0347 (18)	0.049 (2)	−0.0011 (14)	−0.0125 (17)	−0.0188 (17)
C31	0.0269 (16)	0.0278 (16)	0.0194 (15)	−0.0050 (12)	−0.0016 (12)	−0.0062 (13)
C32	0.0262 (17)	0.0302 (16)	0.0276 (17)	−0.0079 (13)	0.0015 (13)	−0.0092 (14)
C33	0.0299 (19)	0.045 (2)	0.048 (2)	−0.0136 (15)	0.0039 (16)	−0.0199 (18)
C34	0.0245 (18)	0.065 (3)	0.042 (2)	−0.0080 (17)	−0.0060 (16)	−0.0217 (19)
C35	0.0294 (19)	0.050 (2)	0.0303 (19)	0.0016 (15)	−0.0084 (15)	−0.0034 (16)
C36	0.0345 (19)	0.0327 (18)	0.0281 (18)	−0.0057 (14)	−0.0003 (15)	−0.0017 (14)
C37	0.0230 (16)	0.0189 (14)	0.0285 (17)	−0.0008 (11)	0.0008 (13)	−0.0033 (12)
C38	0.0250 (17)	0.0363 (18)	0.0278 (18)	−0.0014 (13)	−0.0001 (14)	−0.0083 (14)
C39	0.037 (2)	0.054 (2)	0.0302 (19)	−0.0072 (16)	−0.0008 (16)	−0.0147 (17)
C40	0.037 (2)	0.053 (2)	0.041 (2)	−0.0088 (17)	−0.0122 (17)	−0.0137 (18)
C41	0.033 (2)	0.052 (2)	0.040 (2)	−0.0152 (16)	0.0025 (16)	−0.0121 (18)
C42	0.0347 (19)	0.0400 (19)	0.0266 (18)	−0.0124 (15)	0.0008 (15)	−0.0039 (15)
C43	0.0314 (17)	0.0219 (15)	0.0216 (16)	−0.0006 (12)	−0.0044 (13)	−0.0037 (12)
C44	0.0338 (18)	0.0322 (17)	0.0211 (16)	0.0033 (13)	−0.0037 (13)	−0.0079 (14)
C45	0.058 (2)	0.0327 (19)	0.0253 (18)	0.0140 (16)	−0.0148 (17)	−0.0092 (15)
C46	0.072 (3)	0.0263 (18)	0.039 (2)	−0.0036 (18)	−0.021 (2)	0.0045 (16)
C47	0.058 (3)	0.050 (2)	0.041 (2)	−0.018 (2)	−0.0057 (19)	0.0216 (19)
C48	0.037 (2)	0.044 (2)	0.036 (2)	−0.0037 (16)	−0.0021 (16)	0.0080 (17)
C51	0.0278 (17)	0.0242 (15)	0.0209 (16)	−0.0038 (12)	−0.0001 (13)	−0.0039 (12)
C52	0.0321 (19)	0.0363 (19)	0.041 (2)	0.0053 (14)	−0.0087 (16)	−0.0138 (16)
C53	0.039 (2)	0.045 (2)	0.048 (2)	−0.0053 (16)	−0.0091 (17)	−0.0222 (18)
C54	0.051 (2)	0.0277 (17)	0.038 (2)	−0.0037 (15)	−0.0015 (17)	−0.0155 (15)
C55	0.046 (2)	0.0314 (19)	0.043 (2)	0.0132 (15)	−0.0089 (18)	−0.0109 (16)
C56	0.039 (2)	0.0276 (17)	0.0328 (19)	0.0047 (14)	−0.0076 (15)	−0.0088 (14)

### Geometric parameters (Å, °)

**Table:**

Cd1—Br3	2.5066 (4)	C22—C23	1.370 (5)
Cd1—Br2	2.5156 (5)	C22—H22	0.9500
Cd1—Cl1^i^	2.6479 (4)	C23—C24	1.392 (5)
Cd1—Br1^i^	2.6479 (4)	C23—H23	0.9500
Cd1—Br1	2.6616 (5)	C24—H24	0.9500
Br1—Cd1^i^	2.6479 (4)	C25—C26	1.387 (4)
P1—O1	1.502 (2)	C25—C30	1.400 (4)
P1—C13	1.793 (3)	C26—C27	1.391 (4)
P1—C7	1.794 (3)	C26—H26	0.9500
P1—C1	1.797 (3)	C27—C28	1.373 (5)
P2—O2	1.509 (2)	C27—H27	0.9500
P2—C31	1.788 (3)	C28—C29	1.386 (6)
P2—C25	1.791 (3)	C28—H28	0.9500
P2—C19	1.801 (3)	C29—C30	1.373 (5)
P3—O3	1.500 (2)	C29—H29	0.9500
P3—C51	1.789 (3)	C30—H30	0.9500
P3—C37	1.798 (3)	C31—C32	1.396 (4)
P3—C43	1.802 (3)	C31—C36	1.399 (4)
O4—H41	0.851 (10)	C32—C33	1.376 (5)
O4—H42	0.846 (10)	C32—H32	0.9500
O4—H43	0.848 (10)	C33—C34	1.381 (5)
C1—C6	1.379 (5)	C33—H33	0.9500
C1—C2	1.386 (5)	C34—C35	1.386 (5)
C2—C3	1.371 (6)	C34—H34	0.9500
C2—H2	0.9500	C35—C36	1.380 (5)
C3—C4	1.364 (7)	C35—H35	0.9500
C3—H3	0.9500	C36—H36	0.9500
C4—C5	1.377 (7)	C37—C42	1.389 (4)
C4—H4	0.9500	C37—C38	1.390 (4)
C5—C6	1.391 (6)	C38—C39	1.379 (5)
C5—H5	0.9500	C38—H38	0.9500
C6—H6	0.9500	C39—C40	1.373 (5)
C7—C8	1.385 (5)	C39—H39	0.9500
C7—C12	1.400 (5)	C40—C41	1.389 (5)
C8—C9	1.384 (5)	C40—H40	0.9500
C8—H8	0.9500	C41—C42	1.371 (5)
C9—C10	1.376 (6)	C41—H41A	0.9500
C9—H9	0.9500	C42—H42A	0.9500
C10—C11	1.374 (6)	C43—C48	1.389 (4)
C10—H10	0.9500	C43—C44	1.391 (4)
C11—C12	1.390 (5)	C44—C45	1.390 (4)
C11—H11	0.9500	C44—H44	0.9500
C12—H12	0.9500	C45—C46	1.371 (5)
C13—C18	1.374 (5)	C45—H45	0.9500
C13—C14	1.393 (5)	C46—C47	1.377 (5)
C14—C15	1.384 (5)	C46—H46	0.9500
C14—H14	0.9500	C47—C48	1.387 (5)
C15—C16	1.367 (5)	C47—H47	0.9500
C15—H15	0.9500	C48—H48	0.9500
C16—C17	1.368 (5)	C51—C56	1.392 (4)
C16—H16	0.9500	C51—C52	1.392 (4)
C17—C18	1.390 (5)	C52—C53	1.384 (4)
C17—H17	0.9500	C52—H52	0.9500
C18—H18	0.9500	C53—C54	1.380 (5)
C19—C24	1.391 (5)	C53—H53	0.9500
C19—C20	1.392 (4)	C54—C55	1.368 (5)
C20—C21	1.384 (4)	C54—H54	0.9500
C20—H20	0.9500	C55—C56	1.382 (4)
C21—C22	1.392 (5)	C55—H55	0.9500
C21—H21	0.9500	C56—H56	0.9500
			
Br3—Cd1—Br2	123.627 (16)	C21—C22—H22	119.8
Br3—Cd1—Cl1^i^	108.515 (16)	C22—C23—C24	119.9 (4)
Br2—Cd1—Cl1^i^	108.971 (16)	C22—C23—H23	120.1
Br3—Cd1—Br1^i^	108.515 (16)	C24—C23—H23	120.1
Br2—Cd1—Br1^i^	108.971 (16)	C19—C24—C23	120.0 (3)
Cl1^i^—Cd1—Br1^i^	0.00 (2)	C19—C24—H24	120.0
Br3—Cd1—Br1	110.821 (17)	C23—C24—H24	120.0
Br2—Cd1—Br1	105.115 (17)	C26—C25—C30	119.7 (3)
Cl1^i^—Cd1—Br1	96.353 (14)	C26—C25—P2	122.1 (2)
Br1^i^—Cd1—Br1	96.353 (14)	C30—C25—P2	118.0 (3)
Cd1^i^—Br1—Cd1	83.647 (14)	C25—C26—C27	120.0 (3)
O1—P1—C13	111.51 (14)	C25—C26—H26	120.0
O1—P1—C7	109.07 (15)	C27—C26—H26	120.0
C13—P1—C7	109.32 (15)	C28—C27—C26	120.1 (3)
O1—P1—C1	111.14 (15)	C28—C27—H27	120.0
C13—P1—C1	106.26 (16)	C26—C27—H27	120.0
C7—P1—C1	109.49 (16)	C27—C28—C29	119.9 (3)
O2—P2—C31	108.70 (13)	C27—C28—H28	120.0
O2—P2—C25	113.11 (13)	C29—C28—H28	120.0
C31—P2—C25	109.99 (14)	C30—C29—C28	120.9 (3)
O2—P2—C19	110.91 (14)	C30—C29—H29	119.6
C31—P2—C19	106.83 (14)	C28—C29—H29	119.6
C25—P2—C19	107.12 (14)	C29—C30—C25	119.4 (3)
O3—P3—C51	111.76 (13)	C29—C30—H30	120.3
O3—P3—C37	111.35 (13)	C25—C30—H30	120.3
C51—P3—C37	107.96 (14)	C32—C31—C36	118.9 (3)
O3—P3—C43	107.67 (13)	C32—C31—P2	117.8 (2)
C51—P3—C43	108.35 (13)	C36—C31—P2	123.3 (2)
C37—P3—C43	109.71 (14)	C33—C32—C31	120.2 (3)
H41—O4—H42	107.4 (16)	C33—C32—H32	119.9
H41—O4—H43	107.4 (16)	C31—C32—H32	119.9
H42—O4—H43	108.3 (15)	C32—C33—C34	120.4 (3)
C6—C1—C2	118.9 (4)	C32—C33—H33	119.8
C6—C1—P1	121.0 (3)	C34—C33—H33	119.8
C2—C1—P1	119.4 (3)	C33—C34—C35	120.2 (3)
C3—C2—C1	121.0 (4)	C33—C34—H34	119.9
C3—C2—H2	119.5	C35—C34—H34	119.9
C1—C2—H2	119.5	C36—C35—C34	119.6 (3)
C4—C3—C2	120.3 (5)	C36—C35—H35	120.2
C4—C3—H3	119.9	C34—C35—H35	120.2
C2—C3—H3	119.9	C35—C36—C31	120.6 (3)
C3—C4—C5	119.7 (4)	C35—C36—H36	119.7
C3—C4—H4	120.2	C31—C36—H36	119.7
C5—C4—H4	120.2	C42—C37—C38	118.9 (3)
C4—C5—C6	120.5 (5)	C42—C37—P3	123.1 (2)
C4—C5—H5	119.7	C38—C37—P3	117.9 (2)
C6—C5—H5	119.7	C39—C38—C37	120.1 (3)
C1—C6—C5	119.6 (4)	C39—C38—H38	120.0
C1—C6—H6	120.2	C37—C38—H38	120.0
C5—C6—H6	120.2	C40—C39—C38	120.5 (3)
C8—C7—C12	119.6 (3)	C40—C39—H39	119.7
C8—C7—P1	118.4 (3)	C38—C39—H39	119.7
C12—C7—P1	121.9 (3)	C39—C40—C41	119.9 (3)
C9—C8—C7	120.4 (4)	C39—C40—H40	120.1
C9—C8—H8	119.8	C41—C40—H40	120.1
C7—C8—H8	119.8	C42—C41—C40	119.8 (3)
C10—C9—C8	120.0 (4)	C42—C41—H41A	120.1
C10—C9—H9	120.0	C40—C41—H41A	120.1
C8—C9—H9	120.0	C41—C42—C37	120.8 (3)
C11—C10—C9	120.2 (4)	C41—C42—H42A	119.6
C11—C10—H10	119.9	C37—C42—H42A	119.6
C9—C10—H10	119.9	C48—C43—C44	119.5 (3)
C10—C11—C12	120.7 (4)	C48—C43—P3	123.1 (2)
C10—C11—H11	119.6	C44—C43—P3	117.3 (2)
C12—C11—H11	119.6	C45—C44—C43	120.2 (3)
C11—C12—C7	119.1 (4)	C45—C44—H44	119.9
C11—C12—H12	120.5	C43—C44—H44	119.9
C7—C12—H12	120.5	C46—C45—C44	119.9 (3)
C18—C13—C14	118.6 (3)	C46—C45—H45	120.1
C18—C13—P1	119.8 (3)	C44—C45—H45	120.1
C14—C13—P1	121.6 (3)	C45—C46—C47	120.2 (3)
C15—C14—C13	119.5 (4)	C45—C46—H46	119.9
C15—C14—H14	120.2	C47—C46—H46	119.9
C13—C14—H14	120.2	C46—C47—C48	120.7 (3)
C16—C15—C14	121.5 (4)	C46—C47—H47	119.7
C16—C15—H15	119.2	C48—C47—H47	119.7
C14—C15—H15	119.2	C47—C48—C43	119.5 (3)
C15—C16—C17	119.2 (3)	C47—C48—H48	120.3
C15—C16—H16	120.4	C43—C48—H48	120.3
C17—C16—H16	120.4	C56—C51—C52	118.7 (3)
C16—C17—C18	120.0 (4)	C56—C51—P3	123.3 (2)
C16—C17—H17	120.0	C52—C51—P3	117.6 (2)
C18—C17—H17	120.0	C53—C52—C51	120.9 (3)
C13—C18—C17	121.1 (3)	C53—C52—H52	119.6
C13—C18—H18	119.4	C51—C52—H52	119.6
C17—C18—H18	119.4	C54—C53—C52	119.5 (3)
C24—C19—C20	119.8 (3)	C54—C53—H53	120.3
C24—C19—P2	117.9 (2)	C52—C53—H53	120.3
C20—C19—P2	122.1 (3)	C55—C54—C53	120.2 (3)
C21—C20—C19	119.7 (3)	C55—C54—H54	119.9
C21—C20—H20	120.2	C53—C54—H54	119.9
C19—C20—H20	120.2	C54—C55—C56	120.8 (3)
C20—C21—C22	120.1 (3)	C54—C55—H55	119.6
C20—C21—H21	120.0	C56—C55—H55	119.6
C22—C21—H21	120.0	C55—C56—C51	120.0 (3)
C23—C22—C21	120.5 (3)	C55—C56—H56	120.0
C23—C22—H22	119.8	C51—C56—H56	120.0
			
Br3—Cd1—Br1—Cd1^i^	112.593 (17)	C19—P2—C25—C30	−55.2 (3)
Br2—Cd1—Br1—Cd1^i^	−111.648 (16)	C30—C25—C26—C27	0.9 (4)
Cl1^i^—Cd1—Br1—Cd1^i^	0.0	P2—C25—C26—C27	175.5 (2)
Br1^i^—Cd1—Br1—Cd1^i^	0.0	C25—C26—C27—C28	0.0 (5)
O1—P1—C1—C6	−87.6 (3)	C26—C27—C28—C29	−0.7 (5)
C13—P1—C1—C6	150.9 (3)	C27—C28—C29—C30	0.6 (5)
C7—P1—C1—C6	32.9 (3)	C28—C29—C30—C25	0.3 (5)
O1—P1—C1—C2	82.3 (3)	C26—C25—C30—C29	−1.0 (4)
C13—P1—C1—C2	−39.2 (3)	P2—C25—C30—C29	−175.8 (3)
C7—P1—C1—C2	−157.1 (3)	O2—P2—C31—C32	2.7 (3)
C6—C1—C2—C3	1.0 (6)	C25—P2—C31—C32	−121.6 (2)
P1—C1—C2—C3	−169.2 (4)	C19—P2—C31—C32	122.4 (2)
C1—C2—C3—C4	−0.4 (8)	O2—P2—C31—C36	−174.6 (2)
C2—C3—C4—C5	0.5 (8)	C25—P2—C31—C36	61.0 (3)
C3—C4—C5—C6	−1.3 (7)	C19—P2—C31—C36	−54.9 (3)
C2—C1—C6—C5	−1.7 (6)	C36—C31—C32—C33	−0.7 (4)
P1—C1—C6—C5	168.3 (3)	P2—C31—C32—C33	−178.1 (2)
C4—C5—C6—C1	1.9 (7)	C31—C32—C33—C34	0.3 (5)
O1—P1—C7—C8	−14.2 (3)	C32—C33—C34—C35	0.3 (5)
C13—P1—C7—C8	108.0 (3)	C33—C34—C35—C36	−0.4 (5)
C1—P1—C7—C8	−136.0 (3)	C34—C35—C36—C31	0.0 (5)
O1—P1—C7—C12	168.5 (3)	C32—C31—C36—C35	0.6 (4)
C13—P1—C7—C12	−69.4 (3)	P2—C31—C36—C35	177.9 (2)
C1—P1—C7—C12	46.6 (3)	O3—P3—C37—C42	−179.9 (2)
C12—C7—C8—C9	−0.1 (6)	C51—P3—C37—C42	57.1 (3)
P1—C7—C8—C9	−177.6 (3)	C43—P3—C37—C42	−60.8 (3)
C7—C8—C9—C10	1.2 (6)	O3—P3—C37—C38	−1.2 (3)
C8—C9—C10—C11	−1.1 (6)	C51—P3—C37—C38	−124.2 (2)
C9—C10—C11—C12	0.0 (6)	C43—P3—C37—C38	117.9 (2)
C10—C11—C12—C7	1.0 (5)	C42—C37—C38—C39	0.2 (4)
C8—C7—C12—C11	−0.9 (5)	P3—C37—C38—C39	−178.6 (2)
P1—C7—C12—C11	176.4 (3)	C37—C38—C39—C40	0.1 (5)
O1—P1—C13—C18	−0.9 (3)	C38—C39—C40—C41	−0.8 (5)
C7—P1—C13—C18	−121.6 (3)	C39—C40—C41—C42	1.1 (6)
C1—P1—C13—C18	120.3 (3)	C40—C41—C42—C37	−0.8 (5)
O1—P1—C13—C14	179.7 (3)	C38—C37—C42—C41	0.2 (5)
C7—P1—C13—C14	59.0 (3)	P3—C37—C42—C41	178.9 (3)
C1—P1—C13—C14	−59.0 (3)	O3—P3—C43—C48	178.2 (3)
C18—C13—C14—C15	0.7 (5)	C51—P3—C43—C48	−60.8 (3)
P1—C13—C14—C15	−180.0 (3)	C37—P3—C43—C48	56.9 (3)
C13—C14—C15—C16	−0.6 (6)	O3—P3—C43—C44	0.4 (3)
C14—C15—C16—C17	0.3 (6)	C51—P3—C43—C44	121.4 (2)
C15—C16—C17—C18	0.0 (5)	C37—P3—C43—C44	−121.0 (2)
C14—C13—C18—C17	−0.4 (5)	C48—C43—C44—C45	−0.6 (5)
P1—C13—C18—C17	−179.8 (3)	P3—C43—C44—C45	177.3 (2)
C16—C17—C18—C13	0.0 (5)	C43—C44—C45—C46	−0.9 (5)
O2—P2—C19—C24	27.5 (3)	C44—C45—C46—C47	1.9 (6)
C31—P2—C19—C24	−90.8 (3)	C45—C46—C47—C48	−1.4 (6)
C25—P2—C19—C24	151.4 (3)	C46—C47—C48—C43	−0.2 (6)
O2—P2—C19—C20	−156.4 (3)	C44—C43—C48—C47	1.2 (5)
C31—P2—C19—C20	85.3 (3)	P3—C43—C48—C47	−176.6 (3)
C25—P2—C19—C20	−32.5 (3)	O3—P3—C51—C56	−107.6 (3)
C24—C19—C20—C21	0.1 (5)	C37—P3—C51—C56	15.1 (3)
P2—C19—C20—C21	−175.9 (3)	C43—P3—C51—C56	133.9 (3)
C19—C20—C21—C22	0.6 (5)	O3—P3—C51—C52	64.9 (3)
C20—C21—C22—C23	−0.6 (6)	C37—P3—C51—C52	−172.3 (2)
C21—C22—C23—C24	−0.1 (6)	C43—P3—C51—C52	−53.6 (3)
C20—C19—C24—C23	−0.8 (5)	C56—C51—C52—C53	1.0 (5)
P2—C19—C24—C23	175.4 (3)	P3—C51—C52—C53	−171.9 (3)
C22—C23—C24—C19	0.8 (6)	C51—C52—C53—C54	−0.8 (5)
O2—P2—C25—C26	−107.3 (3)	C52—C53—C54—C55	−0.5 (6)
C31—P2—C25—C26	14.4 (3)	C53—C54—C55—C56	1.7 (6)
C19—P2—C25—C26	130.2 (3)	C54—C55—C56—C51	−1.6 (5)
O2—P2—C25—C30	67.3 (3)	C52—C51—C56—C55	0.2 (5)
C31—P2—C25—C30	−170.9 (2)	P3—C51—C56—C55	172.6 (3)

Symmetry codes: (i) −*x*+1, −*y*+1, −*z*+1.

### Hydrogen-bond geometry (Å, °)

**Table:**

*D*—H···*A*	*D*—H	H···*A*	*D*···*A*	*D*—H···*A*
O4—H41···O1	0.85 (1)	1.62 (1)	2.472 (3)	177 (4)
O4—H42···O2	0.85 (1)	1.63 (1)	2.471 (3)	176 (3)
O4—H43···O3	0.85 (1)	1.63 (1)	2.481 (3)	177 (3)
